# High‐resolution nerve ultrasound and corneal confocal microscopy in taxane‐induced polyneuropathy

**DOI:** 10.1111/ene.16141

**Published:** 2023-11-15

**Authors:** Maria Katz, Hannah Mork, Nazik Baghdasaryan, Lukas Hesse, Kai Wille, Jasmin Treichel, Jeremias Motte, Rafael Klimas, Dietrich Sturm, Peter Dieter Schellinger, Hans‐Joachim Hettlich, Jörg Philipps

**Affiliations:** ^1^ Department of Neurology and Neurogeriatrics, Johannes Wesling Klinikum Minden Ruhr‐University Bochum Minden Germany; ^2^ Clinic for Ophthalmology, Johannes Wesling Klinikum Minden Ruhr‐University Bochum Minden Germany; ^3^ University Clinic for Haematology, Oncology, Haemostaseology and Palliative Care, Johannes Wesling Klinikum Minden Ruhr‐University Bochum Minden Germany; ^4^ Department of Neurology, St Josef‐Hospital Ruhr‐University Bochum Bochum Germany; ^5^ Department of Neurology Agaplesion Bethesda Krankenhaus Wuppertal Germany

**Keywords:** breast cancer, chemotherapy‐induced polyneuropathy, corneal confocal microscopy, high‐resolution nerve ultrasound, nerve conduction studies, paclitaxel, taxane, total neuropathy score

## Abstract

**Background and purpose:**

The role of high‐resolution nerve ultrasound (HRUS) and corneal confocal microscopy (CCM) in the early detection of taxane‐induced polyneuropathy (TIPN) is unclear. The present prospective longitudinal controlled observational pilot study estimates the role of HRUS and CCM in the early diagnosis of TIPN in breast cancer patients.

**Methods:**

Fifteen breast cancer patients receiving paclitaxel and 15 healthy age matched controls were included. Visits before and 3 weeks, 8 weeks and 6 months after treatment included clinical examination, the total neuropathy score, nerve conduction studies (NCS), monocular CCM including corneal nerve fibre length, density and branching and HRUS of bilateral median, ulnar, radial, tibial, peroneal and sural nerves. Patients were compared between different visits and to healthy controls.

**Results:**

Total neuropathy score increased from 2.2 at baseline to 5.8 (*p* < 0.001) at week 8. NCS showed a decreased sensory amplitude in the sural, radial, ulnar and median nerve after 6 months (*p* < 0.001). HRUS revealed a significant increase of cross‐sectional area in the sural nerve (*p* = 0.004), the median nerve (*p* = 0.003) at the carpal tunnel and the ulnar nerve in the forearm (*p* = 0.006) after 6 months. CCM showed no changes at different visits.

**Conclusions:**

Corneal confocal microscopy and HRUS do not detect early signs of TIPN during the paclitaxel treatment period. HRUS and NCS might detect congruent signs of an axonal, predominantly sensory polyneuropathy after 6 months. The clinical examination remains the most sensitive tool in the early detection of TIPN in breast cancer patients.

## INTRODUCTION

Taxane‐induced polyneuropathy (TIPN) is a frequent toxic undesirable effect of taxane chemotherapy; an early diagnosis is increasingly recognized as an interdisciplinary task concerning neurology and oncology [1]. High‐resolution ultrasound (HRUS) of peripheral nerves and corneal confocal microscopy (CCM) can be used in the diagnosis and follow‐up of polyneuropathies [2].

Corneal confocal microscopy is a rapid, non‐invasive technique that quantifies corneal small nerve fibres. It examines the corneal sub‐basal nerve plexus [3] and might detect neurotoxic effects on small nerve fibres.

The role of HRUS and CCM in the early detection of TIPN is unclear. A small number of published studies covering HRUS reported on different types of chemotherapy‐induced polyneuropathy. Lycan et al. [4] found a decrease in sural nerve cross‐sectional area (CSA) in 20 patients with TIPN and an increased median nerve CSA in the carpal tunnel. Pitarokoili et al. [5] reported an increased CSA in 13 patients with oxaliplatin‐induced polyneuropathy, especially at compression sites, as first described by Briani et al. [6] in 15 patients treated with oxaliplatin. TIPN has been characterized as a predominantly length‐dependent sensory neuropathy affecting small nerve fibres [7] with a reduced intraepidermal nerve fibre density [4]. Few studies analysed changes in corneal small nerve fibres in chemotherapy‐induced polyneuropathies using CCM. Bennedsgaard et al. [8] found no affection of corneal small nerve fibres in TIPN, whereas Riva et al. [9] reported a reduction of corneal nerve fibre density (CNFD). Chiang et al. [10] described a reduced corneal nerve fibre length (CNFL) in TIPN. Similar findings have been reported in diabetic neuropathy [11].

The present prospective longitudinal controlled observational pilot study estimates the role of HRUS and CCM in TIPN in breast cancer patients within the first 6 months after starting paclitaxel chemotherapy.

## METHODS

The local ethics committee of the Ruhr University of Bochum, Germany, approved the study protocol compliant with the Declaration of Helsinki (approval number 2020‐609).

### Participants

Participants were adult (>18 years) breast cancer patients treated in the oncology department of the Johannes Wesling University Hospital Minden, Germany, and scheduled for chemotherapy with paclitaxel, or healthy age‐matched controls (relatives or hospital employees). Participants were examined between November 2020 and January 2023.

### Exclusion criteria

Pre‐existing conditions with a potential impact on nerve CSA and CCM (e.g., known diabetes, alcohol abuse, history of polyneuropathy, nerve entrapment syndromes, other peripheral nerve lesions, radiculopathies, peripheral neurosurgical procedures, major trauma to the extremities or end‐stage cancer) led to exclusion.

### Study design

**FIGURE 1:**
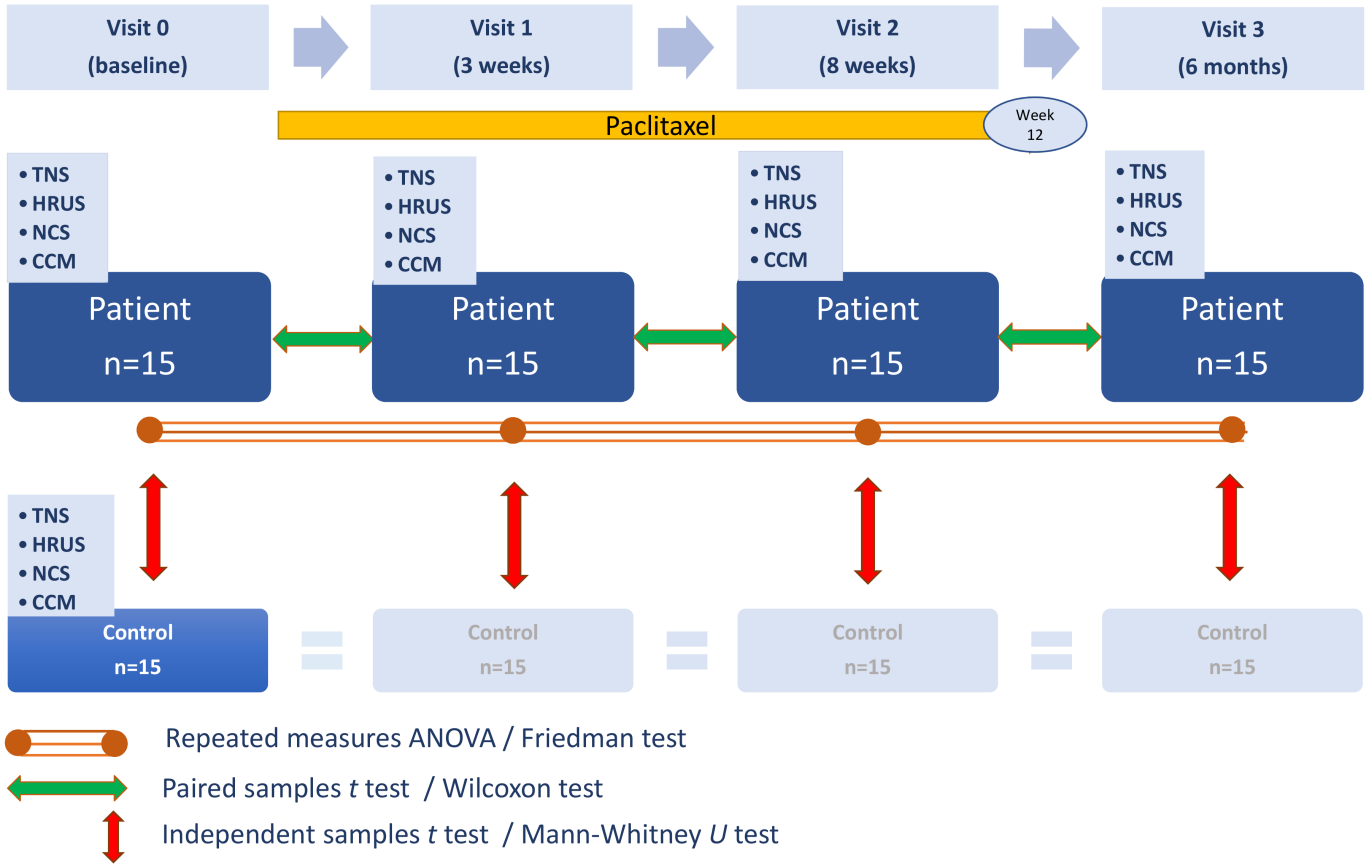
Study design. CCM, corneal confocal microscopy; HRUS, high‐resolution nerve ultrasound; NCS, nerve conduction studies; TNS, total neuropathy score.

Patients were examined at baseline (visit 0) before chemotherapy with paclitaxel and after 3 weeks (visit 1) and 8 weeks (visit 2), both during paclitaxel chemotherapy with a duration of 12 weeks, and 6 months (visit 3) after the beginning of chemotherapy with paclitaxel. Tumour staging and pretreatment with epirubicin and cyclophosphamide (EC) are displayed in Table 1. Healthy controls were examined only once. Nerve conduction studies (NCS), HRUS and CCM were performed by different raters blinded to each other's results. Clinical examination and participant status (patient or control) were not blinded. The study design is summarized in Figure 1.

**TABLE 1:** Demographic and clinical characteristics of individual patients.

Patient number	Age (years)	Height (cm)	Weight (kg)	Breast cancer grading	Previous chemotherapy	Paclitaxel cumulative dose (mg)
1	73	168	68	G2	None	1699.2
2	59	163	80	G2	None	2664.0
3	50	178	74	G2	Epirubicine/cyclophosphamide	1824.0
4	61	160	98	G2	Epirubicine/cyclophosphamide	1900.8
5	46	183	78	G2	None	1929.6
6	66	162	74	G2	None	1728.0
7	63	164	93	G3	Epirubicine/cyclophosphamide	1273.6
8	54	166	89	G2	None	1891.2
9	47	170	85	G1	Epirubicine/cyclophosphamide	1891.2
10	42	175	68	G2	Epirubicine/cyclophosphamide	1699.2
11	50	170	100	G3	Epirubicine/cyclophosphamide	1600.0
12	61	175	78	G2	Epirubicine/cyclophosphamide	1824.0
13	67	168	76	G2	Epirubicine/cyclophosphamide	1728.0
14	52	164	80	G3	Epirubicine/cyclophosphamide	1814.0
15	50	170	65	G2	None	1680.0
Mean (patients) (SD)	56.1 (8.9)	169.1 (6.4)	80.4 (10.7)	n.a.	n.a.	1809.7 (286.9)
Mean (controls) (SD)	52.9 (7.3) *p* = 0.13	170.4 (7.9) *p* = 0.47	76.1 (13.6) *p* = 0.18	n.a.	n.a.	n.a.

*Note*: Means, standard deviation (SD) and *p* values for comparison of patients and healthy controls are indicated.

### Clinical examination

Participants underwent a neurological examination (M. Katz, J. Philipps). Sensory testing for touch and temperature was performed using a 10 g filament (Twin‐Tip®) and a Rydel Seiffer 64 Hz tuning fork for vibratory sensation at both ankles and wrists. The total neuropathy score (TNS) was measured to determine the presence and severity of polyneuropathy. The TNS has been validated for chemotherapy‐induced polyneuropathy [12]. It consists of eight items scored with 0 (normal) to 4 (severely abnormal): sensory and motor symptoms, pinprick and vibration sensation, muscle strength, deep tendon reflexes, tibial nerve compound muscle action potential (CMAP) and sural nerve sensory action potential (SNAP). The sum ranges from 0 (normal) to 32 (severe neuropathy).

### Ultrasound examination

Ultrasound was performed by M. Katz and H. Mork with an Affiniti 50 (Philips, Amsterdam, The Netherlands) device using a 5–18 MHz linear array transducer. Ultrasound pictures and measurements were agreed upon by M. Katz and H. Mork. Measurements were supervised and validated by J. Philipps (board certified neurophysiologist with more than 10 years of experience in nerve ultrasound). For interrater reliability testing, J. Philipps, M. Katz and H. Mork performed 42 blinded CSA measurements of upper limb nerves in seven healthy volunteers before the start of the present study.

The transducer was held at a perpendicular angle to the nerve to obtain the correct CSA. Zoom was not used to avoid alterations in CSA measurement. For CSA measurements in 1/10 mm^2^, nerves were measured in a transverse plane within the inner border of the hyperechoic epineurium using the free‐hand tracer.

### Selection of nerve sites

Each nerve was measured bilaterally at predefined sites. The ulnar nerve was measured at the wrist proximally to Guyon's canal, in the distal third of the forearm, at the ulnar sulcus and in the upper arm at an equal distance between the epicondylus medialis and the axilla. The median nerve was measured at the carpal tunnel, in the distal third of the forearm and in the upper arm. The ulnar and median nerve were traced from the wrist to the proximal upper arm to measure the maximum and the minimum CSA. The intra‐nerve CSA variability (INV), defined as the ratio of the largest to the smallest CSA of one nerve, was calculated for the median and ulnar nerve [13]. The radial nerve was measured in the upper arm in the spiral groove. The sural nerve was measured between the gastrocnemius heads. The peroneal nerve was localized in the popliteal fossa and at the fibular head. The tibial nerve was measured in the popliteal fossa and at the ankle.

### Corneal confocal microscopy

A laser scanning in vivo confocal microscope (Heidelberg Retinal Tomograph 3 with Rostock Corneal Module; Heidelberg Engineering GmbH, Heidelberg, Germany) was used for CCM.

From each monocular examination four representative images showing the sub‐basal nerve plexus in a 400 × 400 μm corneal area (0.16 mm^2^ with a resolution of image in 384 × 384 pixels) were selected. Monocular CCM measures (CNFD, CNFL and corneal nerve branch density, CNBD) were obtained by N. Baghdasaryan and L. Hesse using fully automated software (ACCMetrics V 3.0 image analysis software, University of Manchester, UK).

### Nerve conduction studies

Nerve conduction studies of the median, ulnar, radial, tibial, peroneal and sural nerves were performed bilaterally by J. Philipps and J. Treichel using a Natus Synergy EDX device. The temperature of the extremities was adjusted using warm water if necessary. NCS parameters included the distal motor latency (dmL), CMAP, nerve conduction velocity (NCV) and F‐wave latencies for the median, ulnar, tibial and peroneal nerve. SNAP and NCV were measured for the median, ulnar, radial and sural nerve. In the case of a SNAP amplitude of 0 μV, the sensory NCV was considered as incalculable and excluded from analysis. Reference values of the local electrophysiological laboratory were used.

### Statistical analyses

Based on the difference of means and SD found for the sural nerve CSA in Lycan et al. [4], the minimum sample size to detect a difference with an alpha‐error of 0.001 and a power of 0.9 is 30 pairs for a two‐sided *t* test. Results of bilateral testing (HRUS, NCS) were considered as 30 separate data pairs in 15 patients and 15 controls. Considering four comparisons to the same control group and multiple post hoc tests between different visits, Bonferroni correction was applied and *p* < 0.01 was considered as statistically significant for differences between groups and visits. For regression analyses of all data collected at the four visits, *p* < 0.05 was accepted as significant. Normal distribution of the data was tested using the Kolmogorov–Smirnov test.

Intra‐class correlation coefficients for repeated single measurements with fixed observers and absolute agreement were calculated to assess interrater reliability of HRUS.

The Mann–Whitney *U* test and an independent samples *t* test were used to compare results of clinical, CCM, NCS and HRUS examinations at different visits of patients with the results of one single examination of controls (Figure 1). The Mann–Whitney *U* test was applied to compare breast cancer patient subgroups with and without pretreatment with EC at different visits.

The Friedman test, a repeated measures ANOVA and post hoc paired samples *t* tests or Wilcoxon tests were used to compare results of the TNS, CCM, NCS and HRUS examinations between different visits in the patient group (Figure 1).

Pearson's correlation coefficient was calculated for parametric variables independently of the time of visit in the patient group. Spearman's rank correlation was tested in non‐parametric variables. Correlations were tested for TNS and CSA, INV, cumulative taxane dose and CCM results. Multivariable linear regression models with backward exclusion were used to control for age, weight and height.

IBM SPSS for Windows version 28 (IBM, Armonk, USA) was used for statistical analysis.

## RESULTS

### Demographic characteristics of participants

Twenty patients were scheduled; two patients declined participation. Three patients receiving an additional chemotherapy with carboplatin were excluded. Missing data were not replaced. Demographic characteristics of patients including cumulative chemotherapy dose are shown in Table 1. There were no significant differences with regard to weight, height and age between breast cancer patients at baseline (visit 0) and healthy controls. The sex distribution between groups with four males in the control group was not significantly different (Fisher's exact test, *p* = 0.10).

Complete data (1560 HRUS measurements in 26 bilateral nerve sites) were obtained in nerve ultrasound. Fifty‐nine CCM measurements (each consisting of monocular CNFD, CNBD and CNFL, one missing) were evaluated in the 15 patients at four visits. 94% of HRUS and CCM variables showed a normal distribution. The cumulative paclitaxel dose and the TNS were not normally distributed.

### Interrater reliability

The intra‐class correlation coefficient was 0.73 (0.55–0.85) for M. Katz and J. Philipps; 0.89 (0.81–0.94) for H. Mork and J. Philipps.

### Clinical examination

**FIGURE 2:**
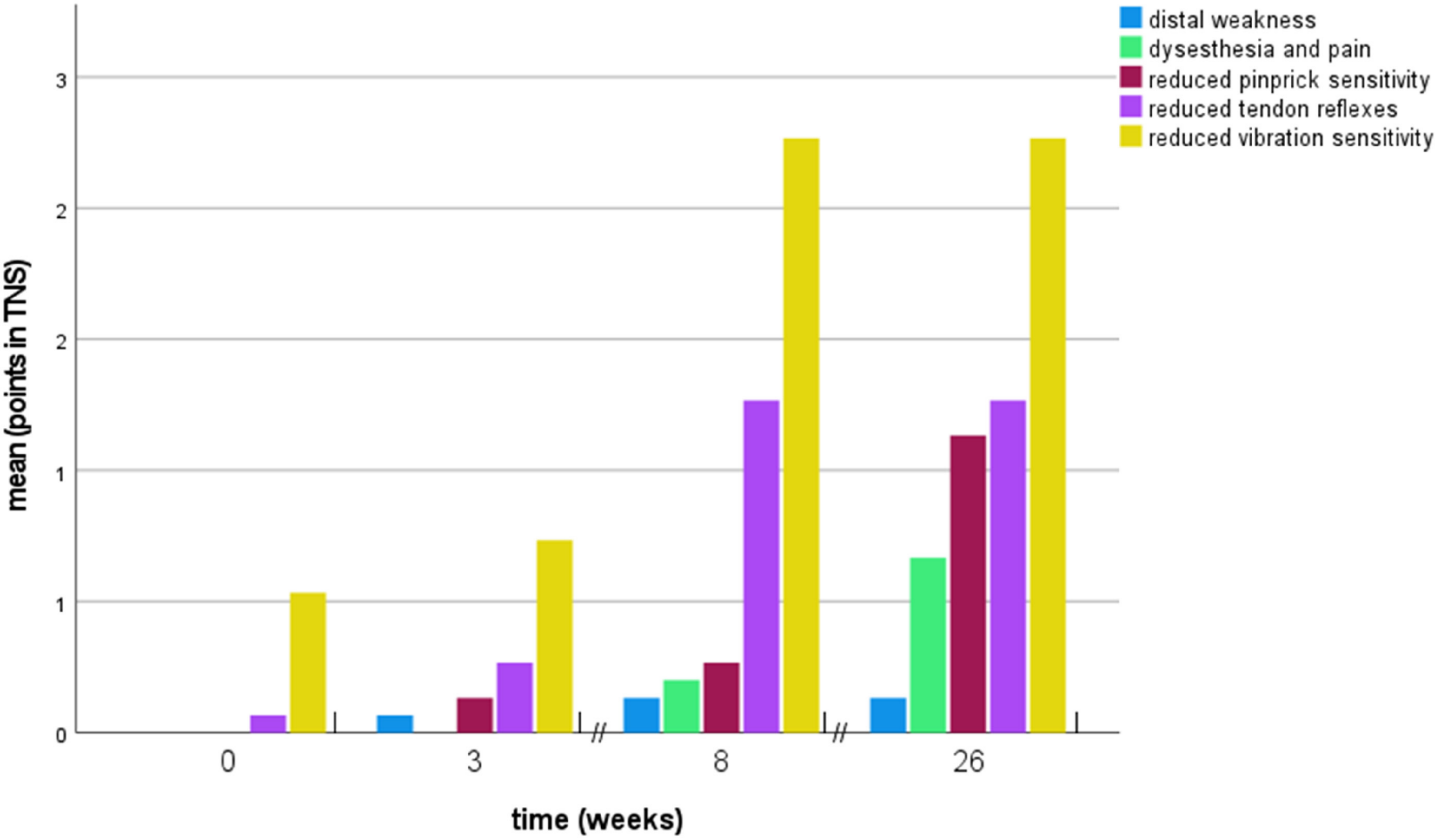
Evolution of clinical symptoms after paclitaxel treatment in breast cancer patients. TNS, total neuropathy score. *n* = 15.

All patients reported neuropathic symptoms or exhibited signs of polyneuropathy relevant to the TNS during the observation period of 6 months. After 6 months, 53% reported sensory symptoms including painful sensations, 86% had a reduced vibration sensation, 73% a reduced pin sensitivity, 66% reported autonomic symptoms, 20% presented with a distal weakness of the toe extensor muscles and 60% had reduced tendon reflexes in the legs. The evolution of clinical symptoms over time is represented in Figure 2.

### Comparison between patients and healthy controls

Total neuropathy score, HRUS (except for the ulnar nerve at the sulcus), NCS and CCM data showed no difference between groups at baseline. The ulnar nerve at the sulcus was excluded from further comparison between groups. There were no differences in patient subgroups with and without pretreatment with EC with one exception: the ulnar nerve SNAP was significantly (*p* < 0.01) higher in the pretreated patients subgroup after 6 months. This was considered as a statistical artifact due to low numbers in both subgroups.

Comparing patients to controls, there was a significantly higher TNS (*p* < 0.001) at week 8 and 6 months in the patient group. NCS showed a significantly reduced radial and sural nerve SNAP, a reduced tibial nerve CMAP and a prolonged dmL after 6 months (*p* < 0.001). HRUS showed a slightly increased sural nerve CSA in patients after 8 weeks (*p* = 0.022) reaching Bonferroni‐corrected significance (*p* = 0.004) after 6 months. The INV of the median (mean 1.9) and ulnar nerve (mean 2.1) was not different between groups at all visits. CCM results were not significantly different between groups at all visits (Table 2).

**TABLE 2:** Total neuropathy score, nerve conduction studies, high‐resolution nerve ultrasound and corneal confocal microscopy in breast cancer patients treated with paclitaxel compared to healthy controls at baseline and after 6 months.

	Controls	Patients (baseline)	Patients (6 months)
TNS (points) (SD)	2.4 (2.0)	2.2 (1.7) *p* = 0.400	10.6 (3.9) ***p* < 0.001**
CNFD (*n*/mm^2^) (SD)	18.9 (7.2)	15.9 (4.5) *p* = 0.169	16.9 (6.9) *p* = 0.440
CNFL (mm/mm^2^) (SD)	9.8 (2.9)	9.2 (2.5) *p* = 0.755	9.1 (2.5) *p* = 0.661
CNBD (*n*/mm^2^) (SD)	27.8 (12.2)	26.2 (12.0) *p* = 0.534	25.1 (13.3) *p* = 0.572
Sural nerve SNAP (μV) (SD)	13.1 (12.3)	13.3 (8.0) *p* = 0.951	4.0 (5.9) ***p* = 0.001**
Sural nerve CSA (mm^2^) (SD)	2.6 (0.7)	2.7 (0.6) *p* = 0.581	3.3 (1.1) ***p* = 0.004**
Radial nerve SNAP (μV) (SD)	8.0 (3.8)	6.6 (1.7) *p* = 0.084	5.5 (2.0) ***p* = 0.003**

*Note*: Means and standard deviations (SD) are indicated. *p* values for independent sample *t* tests compared to controls are indicated. Bold values indicate significant results (*p* < 0.01). *n* = 15.

Abbreviations: CNBD, corneal nerve branch density; CNFD, corneal nerve fibre density; CNFL, corneal nerve fibre length; CSA, cross‐sectional area; SNAP, sensory nerve action potential; TNS, total neuropathy score.

### Comparison between different visits in the patient group

**FIGURE 3:**
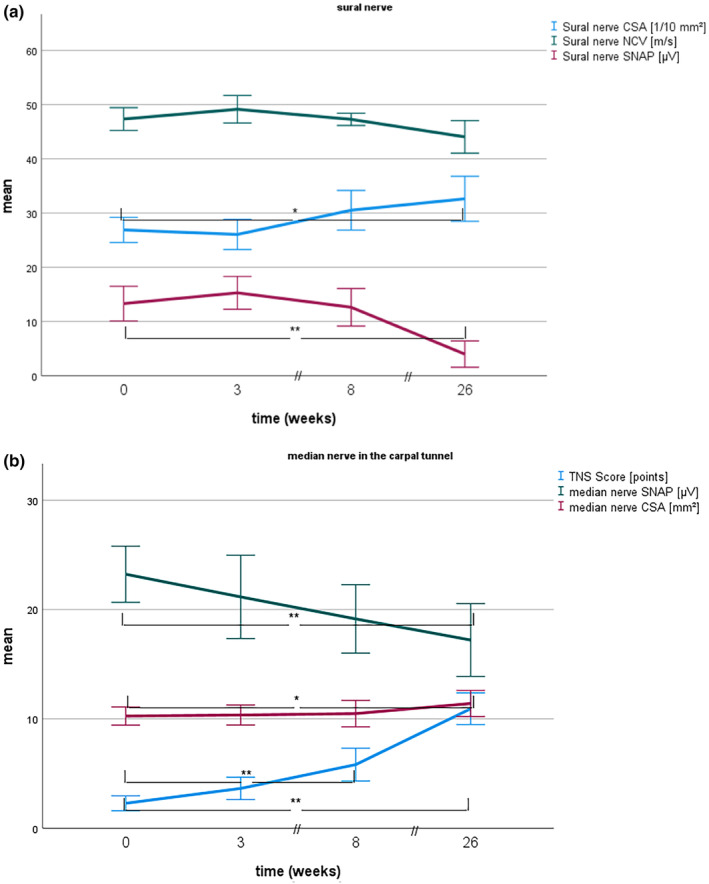
Mean sensory action potential, nerve conduction velocity, cross‐sectional area and total neuropathy score in breast cancer patients treated with paclitaxel at different visits. (a) Sural nerve. (b) Median nerve in the carpal tunnel. CSA, cross‐sectional area; NCV, nerve conduction velocity; SNAP, sensory nerve action potential; TNS, total neuropathy score. *p < 0.01; **p < 0.001 for comparison to baseline values. Error bars indicate 95 % confidence intervals. *n* = 15.

The TNS increased significantly at week 8 (*p* < 0.001). Sural nerve SNAP decreased significantly after 6 months (*p* < 0.001, Table 3, Figure 3a); NCV showed no significant difference. Radial, ulnar and median nerve SNAPs (but not NCV) were significantly reduced after 6 months compared to baseline (*p* < 0.001). Median nerve CSA in the carpal tunnel showed a significant increase after 6 months (*p* = 0.003, Figure 3b). Sural nerve CSA increased slightly at week 8 (*p* = 0.05) and significantly between baseline and 6 months (*p* = 0.004). CSA of the ulnar nerve in the forearm (*p* = 0.006) increased after 6 months. All other nerve sites showed no significant differences between visits. Ulnar and median nerve INVs were not different between visits.

**TABLE 3:** Total neuropathy score, nerve conduction studies and nerve cross‐sectional areas of breast cancer patients treated with paclitaxel at different visits.

	Baseline	3 weeks	8 weeks	6 months
Sural nerve CSA (mm^2^) (SD)	2.7 (0.6)	2.6 (0.7)	3.1 (1.0)	3.3 (1.1) *p* = 0.004
Median nerve CSA, carpal tunnel (mm^2^) (SD)	10.5 (2.2)	10.7 (2.6)	10.5 (3.1)	11.7 (3.0) *p* = 0.003
Ulnar nerve CSA, forearm (mm^2^) (SD)	6.1 (1.6)	7.7 (2.6)	6.8 (2.2)	7.1 (1.8) *p* = 0.006
Sural nerve SNAP (μV) (SD)	13.1 (8.3)	15.3 (7.2)	12.6 (7.7)	4.0 (5.9) *p* < 0.001
Median nerve SNAP (μV) (SD)	23.2 (6.6)	21.1 (9.8)	19.3 (8.1)	17.2 (8.6) *p* < 0.001
Ulnar nerve SNAP (μV) (SD)	22.0 (7.4)	18.3 (10.4)	20.3 (10.7)	13.7 (7.3) *p* < 0.001
Radial nerve SNAP (μV) (SD)	6.7 (1.7)	7.4 (2.3)	5.9 (2.5)	5.3 (1.9) *p* < 0.001
TNS (points) (SD)	2.2 (1.7)	3.6 (2.6)	5.8 (3.8) *p* < 0.001	10.6 (3.9) *p* < 0.001

*Note*: Means and standard deviations (SD) are indicated. *p* values (indicated only if significant with Bonferroni correction, *p* < 0.01) refer to a paired samples *t* test compared to baseline. *n* = 15.

Abbreviations: CSA, cross‐sectional area; SNAP, sensory nerve action potential; TNS, total neuropathy score.

**FIGURE 4:**
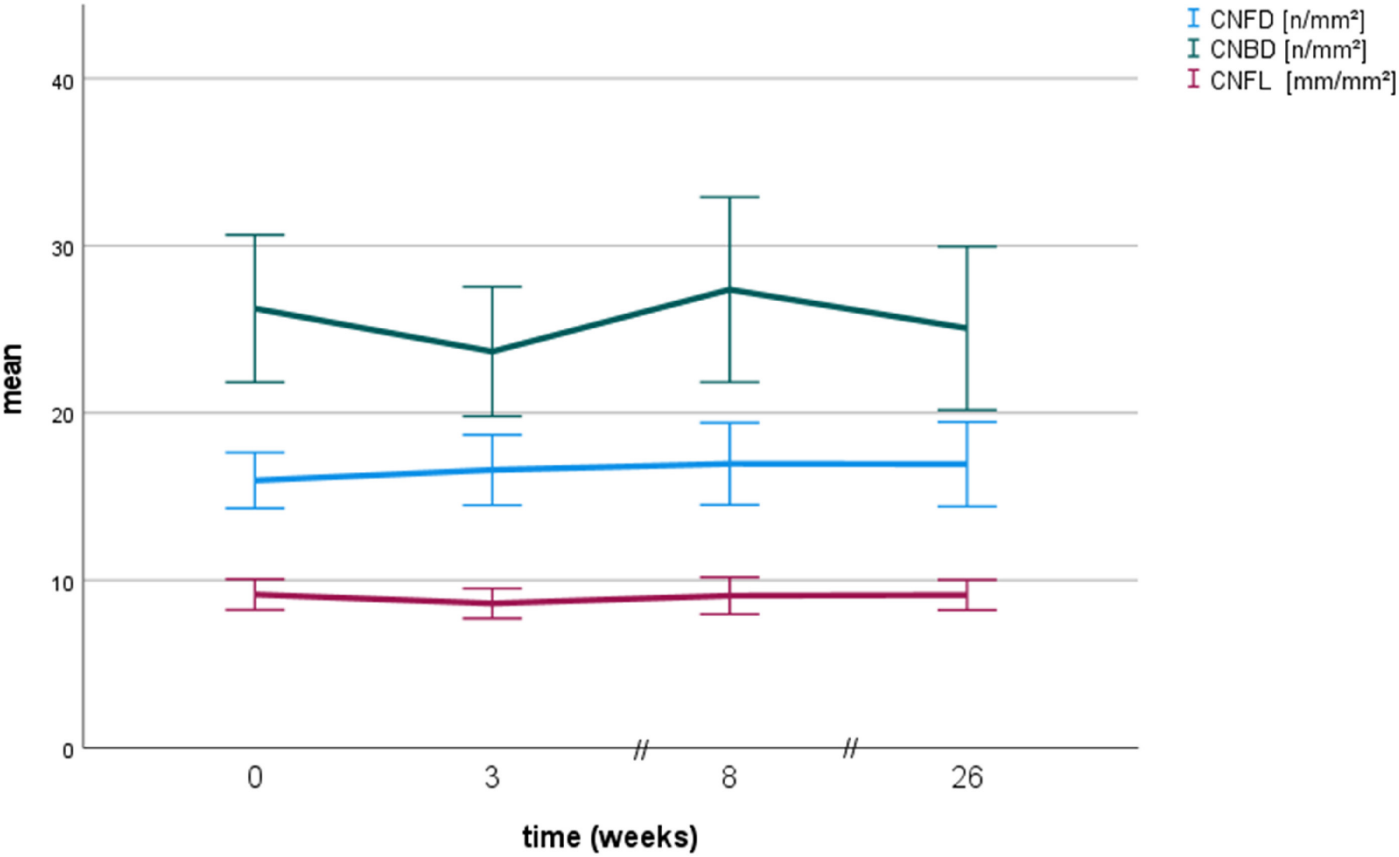
Corneal confocal microscopy results in breast cancer patients treated with paclitaxel at different visits. CNBD, corneal nerve branch density; CNFD, corneal nerve fibre density; CNFL, corneal nerve fibre length. Error bars indicate 95 % confidence intervals. *n* = 15.

Corneal confocal microscopy, NCV and HRUS results at different visits are displayed in Table 2. There were no significant differences between visits for CCM results (Figure 4).

### Correlations

There was a negative rank correlation between the cumulative paclitaxel dose and the sural nerve SNAP (*ρ* = 0.66, *p* < 0.001).

**FIGURE 5:**
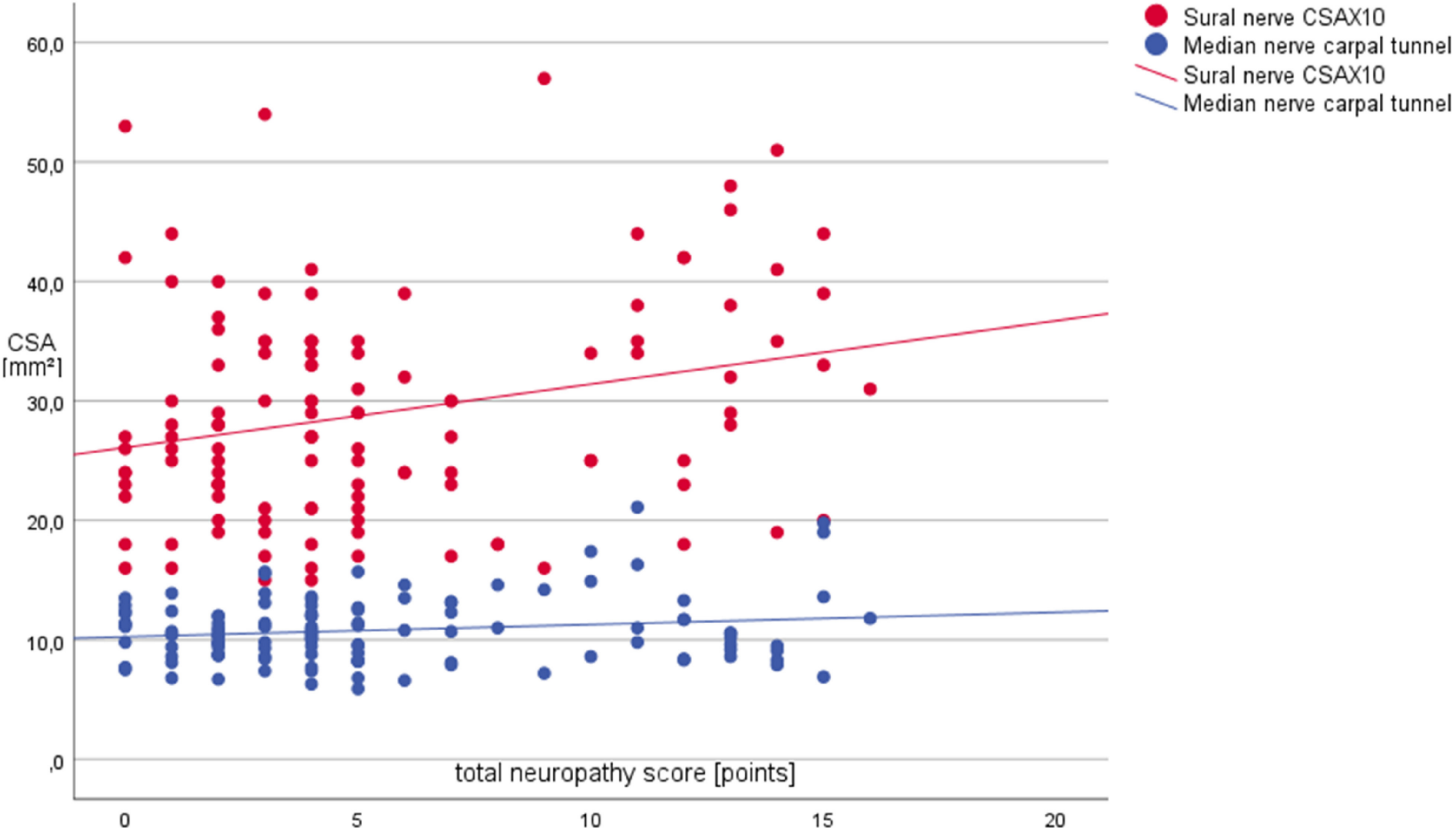
Regression curves of the total neuropathy score and nerve cross‐sectional area in breast cancer patients treated with paclitaxel. CSA, cross‐sectional area. *n* = 15.

No correlation of CCM results with the TNS was found. Age, weight and height were significant predictors of CNFD, CNBD and CNFL. The TNS correlated positively with the CSA of the median nerve in the upper arm (*r* = 0.19, *β* = 0.09, *p* = 0.039); the ulnar nerve in the forearm (*r* = 0.24, *β* = 0.09, *p* = 0.009) and at the elbow (*r* = 0.21, *β* = 0.10, *p* = 0.022); the fibular nerve at the fibular head (*r* = 0.25, *β* = 0.2, *p* = 0.006); the tibial nerve at the ankle (*r* = 0.20, *β* = 0.18, *p* = 0.027); and the sural nerve (*r* = 0.26, *β* = 0.52, *p* = 0.004; Figure 5). The INV of the ulnar and median nerve was not correlated to the TNS. The TNS was the most important predictor of nerve CSA in all nerves, with weight as the second most important factor in the sural nerve, tibial nerve and in the ulnar nerve at the elbow. The TNS showed a significant inverse correlation with the median nerve SNAP (*r* = 0.33, *β* = −0.64, *p* = 0.002) and sensory NCV (*r* = 0.23, *β* = −0.42, *p* = 0.036); the ulnar nerve SNAP (*r* = 0.32, *β* = −0.64, *p* = 0.003); the sural nerve SNAP (*r* = 0.56, *β* = −0.99, *p* < 0.001); and the CMAP of the fibular nerve (*r* = 0.39, *β* = −1.5, *p* < 0.001). The dmL and F‐wave latencies were not correlated with the TNS. The TNS was the most important predictor of the sural and ulnar nerve SNAPs.

## DISCUSSION

The present prospective longitudinal pilot study analyses the capability of HRUS and CCM to detect TIPN in breast cancer patients during and shortly after treatment with paclitaxel in order to prevent further nerve damage [14, 15]. Patients developed symptoms of TIPN detectable as a TNS increase at week 3 reaching a significant level at week 8. This is in line with the literature showing that first symptoms of neurotoxicity occur early in TIPN [15]. A further TNS increase was observed after 6 months, thus 3 months after the end of paclitaxel chemotherapy. This might be explained by the coasting phenomenon. Coasting defined as progressive worsening of neuropathic symptoms after the end of chemotherapy has been described in polyneuropathies associated with platinum‐derived chemotherapies [16] and has been discussed in TIPN as well [17].

No differences of CCM and HRUS measurements were found in patients compared to baseline values and to a healthy control group during the treatment period (12 weeks).

Corneal confocal microscopy (using fully automated software) shows no significant difference to baseline and to healthy controls during the whole observation period of 6 months. The non‐significant decrease in CNBD at week 3 followed by an increase at week 8 occurring during the chemotherapy period is probably not comparable to a regenerative process (CNFL increase) as described by Ferdousi et al. [18] after the end of platinum‐derived chemotherapy. In future, the analysis of further CCM parameters such as the tortuosity coefficient could increase the informative value with respect to regenerative processes [19].

There is no significant correlation between CCM parameters and the TNS. This suggests that CCM does not reflect disease severity in TIPN. This is in line with prior results [8] indicating that CCM is not diagnostic in TIPN. CCM results are correlated with demographic factors as reported in the literature [20]. In the present study, CNFD, CNBD and CNFL are lower than normal values reported in the literature both in healthy controls and in patients. CCM results are difficult to compare between different studies so far, even if the same software is used for analysis. For this reason it would be helpful to develop standards for CCM data analysis and image selection especially in terms of image quality [21].

Nerve ultrasound results suggest that 6 months after start of treatment with paclitaxel a significant increase of CSA can be detected in the sural nerve, the median nerve in the carpal tunnel and the ulnar nerve in the forearm. In the sural nerve, the CSA increase is detectable at week 8 and reaches significance after 6 months. The positive correlation of sural nerve CSA and the TNS points in the same direction. This is in line with the observation of Pitarokoili et al. [5] in oxaliplatin‐associated polyneuropathy, reporting a CSA increase in the leg nerves after 3 months and a tendency towards a CSA increase in compression sites in the upper extremity. Lycan et al. [4] reported an increase of median nerve CSA in the carpal tunnel and a decrease of sural nerve CSA compared to historical controls with a mean sural nerve CSA of 5 mm^2^, most pronounced in patients with remote taxane exposure (>6 months since last taxane dose, mean sural nerve CSA 3.6 mm^2^). In our study, a mean sural nerve CSA of 3.3 mm^2^ 6 months after taxane treatment represents an increase compared to the results of a recent meta‐analysis (Fisse et al. [22, 23], mean 2.4 mm^2^, 95% confidence interval 1.7–3.1 mm^2^), to baseline and to the healthy control group. The healthy control group was introduced to make sure baseline values in the patient group are not affected by paraneoplastic conditions. Baseline values in the patient group were recorded after the end of pretreatment with EC. There was no difference from the control group and in patient subgroups with and without pretreatment, confirming that both substances are not known to cause polyneuropathy [24, 25]. The controlled design represents the clinical situation of a patient with suspected TIPN undergoing HRUS or CCM examination for the first time, thus without individual baseline values. Multivariable analyses suggest the TNS as a significant predictor of sural nerve CSA and show a positive correlation between clinical severity of TIPN and nerve size. It is difficult to compare these results directly with those of Lycan et al. [4], taking into consideration that the sural nerve has a low interrater reliability in nerve ultrasound [26] and that multivariable analyses in small numbers can provide misleading signals despite significant results.

In the present study, the clinical examination revealing progressive distal symmetrical sensory symptoms with predominant hypoesthesia and NCS yielding a decrease mainly in SNAPs and less pronounced in CMAPs with preserved NCVs and F‐wave latencies are compatible with a predominantly sensory, length‐dependent axonal character of TIPN [27]. Slight CSA enlargements in distal nerves and at compression sites combined with a constant INV in HRUS can be interpreted as typical signs of an axonal, non‐inflammatory neuropathy [23]. Paclitaxel hyperstabilizes the microtubules by inhibiting their depolymerization, thus leading to inhibition of mitosis and to alterations in the microtubule structure especially in distal nerve segments. Paclitaxel increases the incidence of swollen and vacuolated mitochondria [28]. This leads to mitchondrial stress, a net energy loss [29] and a disturbed axonal transport, a mechanism observed in axonal neuropathies that are frequently associated with slightly enlarged nerve CSA [30, 31]. The length‐dependent axonal character of TIPN might explain the preservation of CNFD, CNFL and CNBD, as the trigeminal nerve fibres supplying the cornea are relatively short compared to the affected nerves of the extremities.

However, the reduced radial nerve SNAP after 6 months might represent a neuronopathic feature [32], supporting Lycan's hypothesis and animal studies reporting involvement of the dorsal root ganglia in TIPN [33].

In contrast to oxaliplatin, taxanes are linked to both axonal length‐dependent neuropathy and neuronopathy in the literature [32], so the findings of the present study may be compatible with this broader spectrum of tissue damage [29]. A larger study in a multi‐centre design might be able to evaluate the role of HRUS and CCM in TIPN. CCM could be complemented by analysis of corneal inflammatory cells as an additional early marker of neurodegeneration [34].

A major limitation of the present study is the low number of participants. The conclusions should be regarded as hypotheses to be evaluated in studies with larger patient samples. Another limitation is a possible gender bias as only women were included in the patient group. The limitation of follow‐up to 6 months after baseline was a result of the focus on the early phase of TIPN, but constitutes a limitation concerning TIPN in its further course. A strength of the present study is the combined longitudinal and controlled design.

## CONCLUSION

During the paclitaxel treatment period, CCM and HRUS do not seem to detect a reliable early signal of a beginning TIPN. Six months after starting paclitaxel treatment, HRUS and NCS might detect congruent signs of an axonal, predominantly sensory polyneuropathy. The changes seen in the present study suggest an increase of nerve CSA and a decrease of SNAPs. The clinical examination remains the most sensitive tool in the early detection of TIPN in breast cancer patients.

## AUTHOR CONTRIBUTIONS

Maria Katz: Conceptualization; investigation; writing—original draft; methodology; validation; visualization; writing—review and editing; formal analysis; data curation. Hannah Mork: Investigation; writing—review and editing; validation; methodology; conceptualization. Nazik Baghdasaryan: Investigation; writing—review and editing; visualization; methodology; data curation. Lukas Hesse: Investigation; methodology. Kai Wille: Investigation; writing—review and editing; methodology. Jasmin Treichel: Investigation. Jeremias Motte: Writing—review and editing; writing—original draft; supervision. Rafael Klimas: Writing—original draft; writing—review and editing; supervision. Dietrich Sturm: Writing—original draft; writing—review and editing; supervision. Peter Dieter Schellinger: Conceptualization; writing—review and editing; supervision. Hans‐Joachim Hettlich: Conceptualization; writing—original draft; writing—review and editing; supervision. Jörg Philipps: Conceptualization; investigation; writing—original draft; methodology; validation; visualization; writing—review and editing; software; formal analysis; project administration; data curation; supervision. All authors have read and approved the final version of this manuscript.

## CONFLICT OF INTEREST STATEMENT

The authors declare no conflicts of interest.

## INFORMED CONSENT STATEMENT

The participants signed informed consent. The written informed consent included a data processing conformity declaration and consent for publication of anonymized data and images.

## Data Availability

Data are available upon reasonable request to the corresponding author.
